# Research progress of treatment of functional dyspepsia with traditional Chinese medicine compound based on cell signal pathway

**DOI:** 10.3389/fphar.2022.1089231

**Published:** 2023-01-09

**Authors:** Yujiao Song, Defei Yin, Zhenyi Zhang, Lili Chi

**Affiliations:** ^1^ Shandong University of Traditional Chinese Medicine, Jinan, Shandong, China; ^2^ Digestive System Department II, Affiliated Hospital of Shandong University of Traditional Chinese Medicine, Jinan, Shandong, China

**Keywords:** signal pathway, fd, TCM, research progress, treatment

## Abstract

Functional dyspepsia (FD) is the most common clinical gastrointestinal disease, with complex and prolonged clinical symptoms. The prevalence of FD is increasing year by year, seriously affecting the quality of life of patients. The main causes of FD are related to abnormal gastrointestinal dynamics, increased visceral sensitivity, *Helicobacter pylori* (HP) infection, intestinal flora disturbance and psychological factors. A review of the relevant literature reveals that the mechanisms of traditional Chinese medicine (TCM) in the treatment of FD mainly involve the following pathways:5-HT signal pathway, AMPK signal pathway,C-kit signal pathway, CRF signal pathway, PERK signal pathway,NF-κB signal pathway. Based on a holistic concept, TCM promotes gastrointestinal motility, regulates visceral sensitivity and alleviates gastrointestinal inflammation through multiple signal pathways, reflecting the advantages of multi-level, multi-pathway and multi-targeted treatment of FD.

## 1 Introduction

FD is a chronic functional gastrointestinal disorder that presents with a range of symptoms such as postprandial fullness, early satiety, and abdominal pain and cannot be explained by organic, systemic, or metabolic disease. It is defined by Rome IV as a disorder of bowel-brain interaction (DROSSMAN and HASLER, 2016). With reference to the Rome IV diagnostic criteria, FD can be divided into two subtypes, postprandial discomfort syndrome and epigastric pain syndrome, based on the characteristics of the symptoms (DROSSMAN and HASLER, 2016). The pathogenesis of FD is diverse and includes gastrointestinal motility disorders, visceral hypersensitivity, brain-gut axis, gastrointestinal hormone dysregulation, HP infection, genetics and psychosomatic disorders (Douglas, 2016; Tack and Drossman, 2017). At present, most of the clinical treatment is based on HP eradication drugs, gastrointestinal motility drugs, visceral hypersensitivity relief and anti-anxiety and depression drugs, but the efficacy is not satisfactory. Chinese medicine contains a variety of methods such as herbal compounding, herbal monomers and acupuncture for the treatment of FD, which can act synergistically in multiple ways by promoting gastrointestinal dynamics, reducing visceral sensitivity and regulating intestinal flora, with multi-targeted synergistic effects that have certain advantages over the application of Western medicine alone. FD is a psychosomatic disease with complex etiology and closely related to mental and emotional factors, resulting in no specific medication in Western medicine and easy recurrence. Chinese medicine can improve the body’s internal environment, the digestive tract and symptoms of emotional disorders, giving full play to its unique advantages, with few adverse effects and long-term treatment, and is increasingly becoming a research hotspot (Ren et al., 2019; Wang and Pan, 2022). This paper further elaborates the molecular mechanism of Chinese medicine in the treatment of FD based on cell signalling pathways and provides a scientific basis for its exploration of potential therapeutic approaches.

## 2 5-HT signalling pathway

5-HT is a tryptophan-derived neurotransmitter that functions by binding to functionally distinct receptors and is thus involved in many physiological and pathological processes in humans (Jiang et al., 2021). The 5-HT signalling system is widely distributed throughout the body and is responsible for regulating gastrointestinal motility, visceral hypersensitivity, anti-inflammation, regulation of immunity and psychological status. It affects visceral sensitivity by interfering with 5-HT binding to receptors and regulating gastrointestinal secretion and peristalsis (Su et al., et al.). When the gastrointestinal tract is stimulated, intestinal chromophores (EC) release 5-HT and the expression of 5-HT-positive nerve fibres or neurons is enhanced, leading to the activation of multiple neuroactive substances in visceral afferent nerves, causing increased visceral sensitivity and gastrointestinal discomfort (YU et al., 2016). In addition, in the central nervous system, substance *p* (SP) acts as an excitatory transmitter to induce the release of 5-HT, causing visceral hypersensitivity (Teng et al., 2016). 5-HT is a brain-gut neurotransmitter that is found in more than 95% of serum and gastrointestinal tissues. It promotes communication between the brain and the gastrointestinal system, and plays an important role in the brain-gut axis. 5-HT influences gastrointestinal motility by directly affecting smooth muscle and other nerve fibers (Li et al., 2014; Jing et al., 2015). 5-HT concentrations reflect the state of central nervous activity, and reduced function can lead to mood disorders such as anxiety and depression (Duffey et al., 2013). Results of a homozygous study suggest the presence of receptor genes in the chromosomal portion of 5-HT3AR and 5-HT3BR, which can cause depression and schizophrenia (Wu et al., 2015). The pathogenesis of FD is still unclear, but disorders of brain-gut interaction are an important pathogenesis. When 5-HT levels in the central system and gastrointestinal tract are too high, excitotoxicity can be triggered, further causing increased visceral sensitivity and gastrointestinal motility disorders (Stasi et al., 2014; Wauters et al., 2020). The 5-HT1A receptor (HTR1A) can influence neurological activity and mood by regulating dopamine in the brain (Pinna et al., 2021).

In terms of regulating visceral sensitivity, experimental studies have shown that Pitongshu can reduce 5-HT secretion through regulating the 5-HT synaptic pathway, reducing visceral sensitivity and relieving FD symptoms (Chen et al., 2022a). The combination of acupuncture and traditional chinese medicine for children with FD reduces 5-HT levels, promotes gastric motility and effectively improves clinical symptoms (Li et al., 2022a). By observing the effect of Shugan Jianpi Qingre recipe on the expression of 5-hydroxytryptamine1A (5-HT1A) receptor and cyclic phosphatidylinosine effector binding protein (CREB) in the gastric mucosal tissue of FD model rats, we found that it could reduce the expression of 5-HT1A receptor content, increase CREB expression in gastric mucosa, regulate visceral sensitivity, and improve body mass and symptoms in FD rats (Lang et al., 2020). On the other hand, 5-HT is closely related to anxiety and depression, and its reduced function can lead to anxiety, depression and other mood disorders, which can affect the function of other systems, fitting the pathogenesis of FD with liver-depression and spleen-deficiency. Weitong Xiaopi Fang can up-regulate the expression of 5-HT mRNA and protein in FD rats with liver stagnation and spleen deficiency, affect the release and activity of neurotransmitters, and accelerate gastric emptying (Fan et al., 2020). The pharmacological analysis of Liang Shu Wan in the treatment of showed that the FD main signaling pathways include the 5-hydroxytryptamine signaling pathway, and experimental studies showed that Liang Shu Wan could upregulate 5-HT expression in FD rats, improve visceral sensitivity and promote gastrointestinal motility (He et al., 2022). In recent years, studies have shown that structural and functional abnormalities such as duodenal mucosal hypersensitivity can cause gastric hypersensitivity, which is an important mechanism for the occurrence and development of FD, and is affected by the reception and conduction of TRPV1 signals (Barbera et al., 1995; Buschmann et al., 2013; Vanheel et al., 2014). Studies have shown that Xiangsha Liujunzi Decoction can alleviate discomfort by down-regulating local duodenal 5-HT and TRPV1 expression, affecting nerve conduction and relieving visceral hypersensitivity (Zhao et al., 2020).

## 3 AMPK signalling pathway

Adenosine 5′-monophosphate (AMP)-activated protein kinase (AMPK) is found mainly in mitochondria and is a key factor in sensing changes in cellular energy (Garcia and Shaw, 2017). Under physiopathological conditions such as hypoglycemia, hypoxia and local ischemia, AMPK can be phosphorylated by upstream kinases and activated by binding to AMP and ADP. Activated AMPK can regulate various metabolic processes such as cell growth, cell autophagy and apoptosis (Villanueva-Paz et al., 2016; Wang et al., 2016). Autophagy is an important pathway for recycling through lysosomal removal of misfolded proteins, damaged organelles, etc (Bravo-San Pedro et al., 2017). Mitochondrial autophagy is specific macroautophagy that selectively degrades damaged or senescent mitochondria for recycling and is susceptible to the intracellular environment (Bharath et al., 2021; Chen et al., 2022b). In AMPK knockout mouse embryonic fibroblasts, ULK1 is not activated, autophagy is inhibited and damaged mitochondria accumulate. Studies have confirmed that AMPK-induced phosphorylation of ULK1 plays an important role in autophagy (Li and Chen, 2019). Increased levels of AMPK phosphorylation inhibit the activity of phosphorylated target of rapamycin (p-mTOR) proteins, leading to autophagy (Li and Chen, 2019). Gastrointestinal motility disorders are an important part of the pathogenesis of FD, and their main pathogenesis is related to the interstitial cells of Cajal (ICC) or ghrelin (SANGER and PASRICHA, 2017). Ghrelin is a brain-intestinal peptide that promotes gastric acid secretion and accelerates gastric emptying, and mTOR in gastric sinus tissue has been found to ghrelin expression was found to be affected by mTOR in gastric sinus tissue (Kang et al., 2017). Ghrelin was found to enhance phosphorylation of AMPK and its targets in the hypothalamus, suggesting that the promotion of FD gastrointestinal motility by Ghrelin may be related to the AMPK signalling pathway (STEVANOVIC et al., 2012). Slow wave potentials are generated by the ICC and are involved in the signalling of gastrointestinal neurotransmitters. Abnormalities in the number and structure of ICC cause gastrointestinal motility disorders, which may be associated with excessive ICC autophagy (Huang et al., 2017; Sanders, 2019; Zhang et al., 2021). Thus FD pathogenesis may be related to the AMPK-mTOR signalling pathway.

Studies have shown that Pingwei Capsules, based on Pingwei San, regulate gastrointestinal motility disorders in FD with liver depression and spleen deficiency through AMPK signaling pathway-mediated ICC autophagy. (Mao et al., 2021). Tang Lei found that under the same intervention conditions, FD rats showed the same trend of changes in daily behavioural status, gastrointestinal motility and Ghrelin expression, and the opposite trend of changes in mTOR expression, indicating its negative regulatory effect and the negative regulation of mTOR by AMPK. The mechanism of action may be related to the promotion of AMPK phosphorylation, resulting in a decrease in mTOR and an increase in Ghrelin (Tang et al., 2020).

## 4 C-kit signalling pathway

C-kit is a proto-oncogene product and a specific marker of gastrointestinal ICC. Downregulation or loss of the kit signalling pathway results in the transformation of ICCs to smooth muscle-like cells (Tang et al., 2020). It binds to the ligand stem cell factor (SCF) and initiates the SCF/C-kit signalling pathway, which plays an important role in maintaining ICC proliferation and differentiation and is closely related to gastrointestinal motility (Bashamboo et al., 2006; Zhang et al., 2017). Abnormalities in the SCF/C-kit signalling pathway can trigger specific expression of genes that regulate ICC proliferation, differentiation and development in the gastrointestinal tract, leading to abnormal slow-wave rhythms that reduce smooth muscle motility and slow down the dynamics of the gastrointestinal tract, thereby inducing gastrointestinal motility dysfunction-related diseases such as FD (Chai et al., 2017).

Chen Qiao used the “Using the acu-points of shu to regulate the center of shu” method to treat rats with gastrointestinal motility disorders to observe the expression of SCF and its C-kit protein and mRNA. Studies have shown that “Using the acu-points of shu to regulate the center of shu” acts on the Back-Shu point through acupoint pointer therapy to generate the corresponding bioelectric signal, and through the operation of the meridian Qi, the extracellular signal is transmitted and acts on the SCF/C-kit signaling pathway to improve the mechanism of gastrointestinal dynamics (Chen et al., 2019). By observing the gastric ICCs and their SCF/C-kit signaling pathways in FD rats, Deng found that SCF and C-kit decreased significantly in FD model rats compared with normal rats, SCF and C-kit increased in the low, medium and high dose groups of Aurantii Fructus Immaturus compared with the model group, so Aurantii Fructus Immaturus could promote the proliferation of ICCs through upregulating the expression of SCF and C-kit to promote gastric motility (Deng et al., 2018). It was found that mild moxibustion at the Zusanli could promote ICC proliferation by upregulating SCF and C-kit expression through the SCF/C-kit pathway, resulting in a significant increase in gastric emptying rate to improve gastrointestinal motility (Han, 2020). By observing the effects of magnolol on digestive fluid secretion and gastrointestinal motility in FD mice, Wang found that magnolol could upregulate SCF and C-kit expression and improve symptoms in FD mice, and that gastrointestinal hormones and gastrointestinal motility were significantly improved (Wang et al., 2020a). Based on the SCF/C-kit signaling pathway, Weichangshutai granules increased the expression of SCF and C-kit in the gastric sinus of mice and promoted the proliferation of ICCs, thus improving gastrointestinal motility and effectively improving their symptoms (Wang et al., 2022).

## 5 CRF signalling pathway

CRF is a 41-amino acid neuroendocrine peptide that is widely distributed in the central nervous system and peripheral tissues such as the gastrointestinal tract. CRF is found in the hypothalamus, paraventricular nucleus and amygdala in the central system and in the adrenal glands and gastrointestinal tract in the peripheral system (Giannogonas et al., 2016). CRF is a key regulator of the stress response, and various stressors can stimulate the pituitary-adrenal axis by inducing CRF release, and can act as a neuromodulator of behavioural and autonomic nervous system activity, regulating visceral function under stressful conditions, and is closely related to gastrointestinal dynamics and increased visceral sensitivity (Peng and Liu, 2007; Larauche et al., 2009). Animal studies have shown that intracerebral injection of CRF or related peptides can inhibit gastric emptying and gastric motility, and Tache found that intracerebellar CRF injection into the medullary pool of the rat cerebellum can similarly inhibit gastric emptying of liquid food in rats (Taché et al., 1987; Tache et al., 2018). The CRF receptor family includes two G-protein-coupled receptors, corticotropin releasing factor-receptor 1(CRF-R1) and corticotropin releasing factor-receptor 2(CRF-R2), which in terms of mood regulation, bind mainly to CRF-R1, which enhances anxiety-depression-like behaviours, and to CRF-R2, which inhibits anxiety-depression-like behaviours (Jezova et al., 1999). Stress can trigger alterations in the brain-gut axis, which can ultimately lead to the development of Gastrointestinal diseases such as FD (Konturek Peter et al., 2011). A study of FD patients showed that CRF levels were significantly higher in FD patients than in controls, and higher CRF levels were associated with higher anxiety-depression scores, suggesting that CRF participates in the development of FD and has an impact on anxiety-depression (Li et al., 2016). Studies have shown that CRF can cause a low inflammatory state in FD patients by regulating the levels of mast cells and cytokines (Shin-Ichiro et al., 2019).

By observing the distribution and expression of peripheral duodenal CRF and CRF-R1 in the central nervous system of FD rats, Zhu found that CRF expression was up-regulated in FD rats and that Jianpiliqi Fang could inhibit CRF expression levels and participate in brain-gut interaction, thus treating FD (Zhu et al., 2020). Li showed that the expression of CRF in the gastric sinus of FD rats was higher than that of the normal group, and the expression of urocortin 2 (UCN2) was lower than that of the normal group, and Xiangsha Liujunzi Decoction could reduce the level of CRF in the gastric sinus of FD rats with spleen deficiency, and the effect was more significant at higher doses, which could increase the expression of UCN2 mRNA and protein, improve gastric emptying rate and promote gastric motility (Li et al., 2022b). 5-HT is an important neurotransmitter in the gastrointestinal tract that is regulated by the hypothalamic CRF for synthesis and release and binds to specific receptors (Nakade et al., 2007; Qin et al., 2019). Xiangsha Liujunzi Decoction can reduce 5-HT secretion by reducing CRF levels in FD rats. It was found that Sinisan could reduce the expression of CRF in the central system and peripheral tissues of FD model rats, and also reduce the expression level of CRF-R1 in the duodenum to regulate brain-gut interaction (Zhu, 2020).

## 6 PERK signalling pathway

Endoplasmic reticulum stress (ERS) and its mediated unfolded protein response (UPR) play an important regulatory role in the cellular response to external stimuli, controlling cell survival and extinction through the expression of apoptosis and autophagy-related genes (Wu et al., 2016; Hetz and Papa, 2018). Appropriate endoplasmic reticulum stress is beneficial to intracellular homeostasis, but excessive endoplasmic reticulum stress can lead to associated apoptosis. The ERS activates the unfolded protein response (UPR) pathway, which is mainly activated by PKR-like ER kinase (PERK), inositol-requiring enzyme 1(IRE1), and acti-vating transcription factor 6 (ATF6), three endoplasmic reticulum transmembrane sensing proteins (Banerjee et al., 2016). PERK is the first activation pathway in response to endoplasmic reticulum stress (Vandewynckel et al., 2015). Under physiological conditions, all three pathway initiator proteins are bound to endoplasmic reticulum molecular chaperone regulatory protein 78 (GRP78) in the lumen of the endoplasmic reticulum in an unactivated state. When stress occurs, the three proteins dissociate from GRP78, accelerating the degradation of misfolded proteins, reducing endoplasmic reticulum stress and promoting cell survival. If the endoplasmic reticulum is in a state of continuous stress and PERK is chronically activated then cell viability will be impaired (Ye et al., 2013). The interconnected nerve fibres of ICC and smooth muscle cells jointly regulate gastrointestinal motility, and the presence of autophagy in their cells, causing a reduction in their number, may be an important cause of FD gastrointestinal motility disorders (Jan et al., 2009; Wang and Jiang, 2009; Ji et al., 2013).

By observing the expression of PERK protein in the PixuⅠFormula, FD rats and the treatment of FD with Spleen Deficiency Formula 1, Lv showed that the expression of PERK protein was elevated in model FD rats compared with the normal group, and after the drug intervention with Spleen Deficiency Formula 1, the expression of each PERK protein was reduced compared with the model group, which could improve the cellular UPR phenomenon (Lv et al., 2017). Zhou used a cycle-length modelling method to show that the degree of ERS occurrence in this model FD rats was persistent, and whether ERS occurred in the gastric tissues of FD rats was confirmed by comparing the PERK expression. The experimental results showed that the PERK level was higher in the model FD rats compared with the normal group, and the PERK decreased significantly after the drug treatment with Chaihushugan Powder was given, indicating that Chaihushugan Powder promotes gastric motility through the ERS (Zhou, 2018). Separation of PERK from GRP78 leads to phosphorylation of Ser51 of elF2α, or p-elF2α, thus reducing the burden of protein synthesis by the endoplasmic reticulum (Heather et al., 2000). It was found that p-elF2αprotein expression was elevated in FD model rats, indicating that elF2αwas heavily phosphorylated in the gastric tissues of FD rats. After Chaihushugan Powder intervened in FD rats, p-elF2αexpression was reduced, indicating that elF2α phosphorylation was inhibited, from which it can be deduced that Chaihushugan Powder has an inhibitory effect on the PERK/elF2αpathway, suppressing endoplasmic reticulum stress and promoting gastric motility (Zhou, 2018).

## 7 NF-κB signalling pathway

NF-κB is a group of nuclear protein factors that regulate the expression of a wide range of genes and play an important role in the regulation of cell proliferation, differentiation, inflammatory response and apoptosis in the body (Yarandi and Christie, 2013). NF-κB family includes five subunits, including p65 and p50, and the typical NF-κB is a dimer composed of p50 and p65. p65 is present in the resting state with inhibitor kappaB(IκB) and exists in the cytoplasm as a homo- or heterodimeric form in an inactive state. When it receives pathological stimuli, it activates the apoptotic signaling pathway (Cramer et al., 2012). Previous studies on FD have mainly been related to abnormalities in gastric structural function; recent studies have shown a close association between inflammatory cell infiltration in the duodenum, elevated local inflammatory factors and impaired expression of barrier function-tight junction proteins (Chang et al., 2017). toll-like receptor 9 (TLR9) and toll-like receptor 4 (TRL4) play an important role in the stress-induced inflammatory response, which induces disease.

It was found that NF-κB expression was elevated in gastric smooth muscle cells of model FD rats, and after drug intervention with Chaihushugan Powder, NF-κB expression was significantly reduced in FD rats, indicating that Chaihushugan Powder could inhibit excessive apoptosis of gastric smooth muscle tissue cells by inhibiting the activation of NF-κB signaling pathway in order to promote gastric motility and improve FD symptoms (Shang, 2017). By observing the gastrointestinal motility and NF-κB p65 expression in the hypothalamus tissue of FD rats with liver-depression and spleen-deficiency type and the treatment of liver-depression and spleen-deficiency type with Chaizhu Liweiyin, Fan found that the NF-κB p65 protein and gene expression in the hypothalamus tissue of FD rats in the model group was elevated, and different dose groups of the formula could reduce the NF-κB p65 expression to different degrees (Fan et al., 2021). TLR9 and TRL4 play an important role in stress-induced inflammatory responses. TLR9 was elevated in the duodenum, and its level decreased significantly after treatment with Chinese herbal gavage. NF-κB is the most important signaling molecule downstream of TLR9, and activated TLR9 activates NF-κB, which promotes the expression of inflammatory factors such as TNF-α (Chang et al., 2018). Chaihu Shugan San can not only inhibit apoptosis by inhibiting the activation of NF-κB signaling pathway, but also have an inhibitory effect through PERK/elF2α pathway, inhibiting endoplasmic reticulum stress and promoting gastric motility. When NF-κB is stimulated, a detached IκB in NF-κB enters the nucleus to bind to DNA and regulate the inflammatory response. Electroacupuncture intervention in FD model rats resulted in a decrease in duodenal TLR4 expression and a decrease in inflammatory factors such as IL-6 and TNF-α, which inhibited the inflammatory response and repaired the mucosal barrier, thereby reducing FD-related symptoms (Wang et al., 2020b).

## 8 Other signalling pathways

C-type natriuretic peptide (CNP) binds specifically to natriuretic peptide receptor B (NPR-B) on smooth muscle cell membranes, which activates pGC, causing an increase in the intracellular second messenger cyclic guanosine monophosphate (cGMP). cGMP inhibits the voluntary contraction of smooth muscle (Gower et al., 2000; Guo et al., 2003). Zhong observed the CNP and cGMP contents and NPRB expression in gastric smooth muscle in FD rats after the intervention of Sijunzi Decoction, and showed that the CNP and cGMP contents decreased and NPR-B expression decreased after the intervention of Sijunzi Decoction, indicating that Sijunzi Decoction could regulate the CNP-NPR-B-cGMP signalling pathway and increase gastric emptying capacity to normalize it (Zhong et al., 2017). Huang found that after 2 weeks of treatment with Shuwei Decoction in FD rats, the expression of pGC protein, CNP protein and mRNA was significantly reduced compared to the model group to inhibit the CNP/pGC signalling pathway and restore gastric motility (Tang et al., 2017). MEK signalling pathway is regulated by extracellular signal-regulated protein kinase (ERK) protein kinase (ERK), c-junnh2-terminal kinase (JNK) and p38, a cascade of three protein kinases involved in cell proliferation, differentiation and apoptosis (Kang et al., 2018). The effect of electroacupuncture on the MEK/ERK1/2 signalling pathway in FD rats was observed by Kang who used tail-clamping stimulation with irregular diet to model FD rats. The studies found that MEK, ERK1/2 and their phosphorylated proteins p-MEK and p-ERK1/2 proteins were elevated in FD model rats, and that the expression of MEK and ERK1/2 and their phosphorylation levels were inhibited by electroacupuncture at the Zusanli. Prostaglandin E2 (PGE2) is a mucosal protective endogenous factor, upon binding to its receptor, elevates intracellular cyclic adenosine monophosphate (cAMP) and has a regulatory effect on the opening of tight junction proteins and epithelial paracellular pathways (Su et al., 2008; Tan et al., 2017). It was found that the expression of PGE2 and cAMP decreased in FD model rats, and that Jianpi Yiqi Recipe could up-regulate PGE2-cAMP expression and improve the structure and function of duodenal tight junction protein in FD rats, which is one of the mechanisms for the treatment of FD (Zhao et al., 2019).

Information transfer between cells is a fundamental law of life activity. Molecular signals stimulate the cell membrane or intracellular receptors, and the ligand binds to the receptor, initiating a cascade of intracellular signaling, which transmits extracellular signals to the nucleus, altering the transcriptional activity of target genes and ultimately generating a series of cellular responses that cause physiological reactions in the body (Huang and Zhu, 2005; Yang and Guo, 2015). The pathogenesis of FD is related to a variety of signalling pathways. With the continuous progress of molecular biology research, the understanding of FD-related cell signalling pathways has been deepened, and the mechanism of Chinese medicine intervention in FD has gradually become a research hotspot in recent years. The 5-HT signaling pathway, AMPK signaling pathway, C-kit signaling pathway, CRF signaling pathway, PERK signaling pathway and NF-κB signaling pathway are the pathways of FD treatment in Chinese medicine. The 5-HT signaling pathway and CRF signaling pathway play an important role in mediating visceral hypersensitivity. AMPK, C-kit and PERK signaling pathways are closely related to mediating cellular autophagy and regulating gastric motility. NF-κB signaling pathway is mainly related to mediating inflammatory response.

FD is a psychosomatic disorder and is closely related to the spleen and stomach. The liver is the master of drainage, which affects the spleen and stomach’s ability to raise and lower turbidity, so the basic clinical principle is to clear the liver and relieve depression, regulate qi and strengthen the spleen. Chinese medicine has a long history of treatment for FD, with the advantages of multi-component, multi-target and multi-level, and combined with modern medicine multiple cell signaling pathways, it plays a unique advantage in reducing visceral sensitivity, regulating gastric dynamics and reducing inflammatory response.

Current research related to the prevention and treatment of FD in Chinese medicine reflects some of the shortcomings, and there is still a gap with Western medical research: (1) Due to the insufficient level of research in Chinese medicine, too much research is focused on single and compound herbs, and research on single herbs is slightly thin. (2) Lack of multi-pathway research on the prevention and treatment of FD by Chinese medicine compounding, which cannot better exploit the therapeutic advantages of Chinese medicine with multi-pathways and multi-targets. (3) Chinese herbal compounding has not been analysed for specific components of action in the course of the study. (4) Research on the treatment of FD with TCM is still dominated by basic research and there is a need for high quality randomized controlled clinical trials to provide assurance on the clinical role of TCM. In summary, there is still much room for exploring the mechanism of TCM in the treatment of FD. Future research should be based on TCM theory as a guide, discriminating evidence, making full use of modern medicine’s advanced experimental instruments and science and technology to summarize research involving all pathways of a single compound formula, to comprehensively, thoroughly, systematically and objectively explore the mechanism of TCM in the treatment of FD, and to provide a more favourable evidence-based medical basis for the clinical application and promotion of TCM.
